# Impact of Correlated Color Temperature on Visitors’ Perception and Preference in Virtual Reality Museum Exhibitions

**DOI:** 10.3390/ijerph20042811

**Published:** 2023-02-05

**Authors:** Na Yu, Yue Lv, Xiaorong Liu, Shuai Jiang, Huixuan Xie, Xiaofan Zhang, Ke Xu

**Affiliations:** 1 College of Furnishing and Lndustrial Design, Nanjing Forestry University, Nanjing 210037, China; 2 Co-Innovation Center of Efficient Processing and Utilization of Forest Resources, Nanjing Forestry University, Nanjing 210037, China; 3 College of Art and Design, Nanjing Forestry University, Nanjing 210037, China; 4 Exhibition Department, Nanjing Museum, Nanjing 210095, China

**Keywords:** psychophysiology variables, correlated color temperature, perception, preference, museum exhibition, sex and major discrepancies

## Abstract

From the perspective of psychophysiological evaluation, this paper provides a theoretical reference for the lighting settings of museums. In order to study the impact of correlated color temperature (CCT) on visitors’ perception and preference in museum exhibitions, an experiment was conducted in the ergonomics laboratory of Nanjing Forestry University. We invited 50 participants to visit the virtual reality museum exhibitions with different CCTs, built by Autodesk 3D’s Max 2017. Specific psychophysiology variables—eye movement, electrodermal activity (EDA), and heart rate variability (HRV)—and the perception and preference of participants were collected. The results indicated that the association of CCT with eye movement, HRV, and some perceptual dimensions was significant. Under high illumination conditions with different CCTs, the pupil diameter and warmth decreased with the increase in CCT, but the comfort and pleasure scores increased first and then decreased. The CCT scenes sorted by LF/HF ratio from high to low were 4500 K, 6000 K, and 3000 K, which was consistent with the results of preference ranking. The LF/HF ratio showed significant sex differences and major discrepancies.

## 1. Introduction

Currently, the research focus of museum exhibition design has gradually shifted from the study of objects to the study of people. With the development of the cultural industry and the improvement of living standards, the number of visitors to the museum is increasing day by day, and their demand is rising for a better experience. The visitors’ experience is affected by many elements, and the lighting is one of the most important aspects of museum exhibition design. Most museum lighting studies focused on energy optimization and visual effect [1]. Its complexity lies in not only slowing down the aging of cultural relics but also meeting the visual experience of the visitors [2]. There are relevant specifications for the lighting conditions set based on the protection of cultural relics, while the lighting conditions guided by the visual experience of the visitors still have different standards than in previous studies due to the diversity of influencing factors [3].

Francesco Leccese et al. presented lighting arrangements with different lighting contrast ratios to observers in order to investigate the trends of personal preference. The results of the surveys pointed out that the observers preferred more uniform and relaxing lighting scenarios [4]. Haiwen Gao et al. explored the influence of different lighting modes (general lighting, accent lighting, and mixed lighting) and correlated color temperature (CCT) changes on the observers’ psychology. The research showed that only accent lighting was better with CCT at 4000 K (Kelvin, the measurement unit of CCT), and the mixed lighting effect was better with CCT at 2700 K [5]. CCT, widely used to describe the color information of lights, is a parameter in optical radiation measurement about chromaticity, not a subjective measure of visual perception [6]. Francesca Feltrin et al. found that the CCT of the lighting was the main factor affecting the painting’s appearance, and the perceived brightness increased along with the CCT [7]. However, there is still no unified conclusion on people’s perception and preference of CCT in the previous literature, especially in museums [8,9,10].

**Figure 1:**
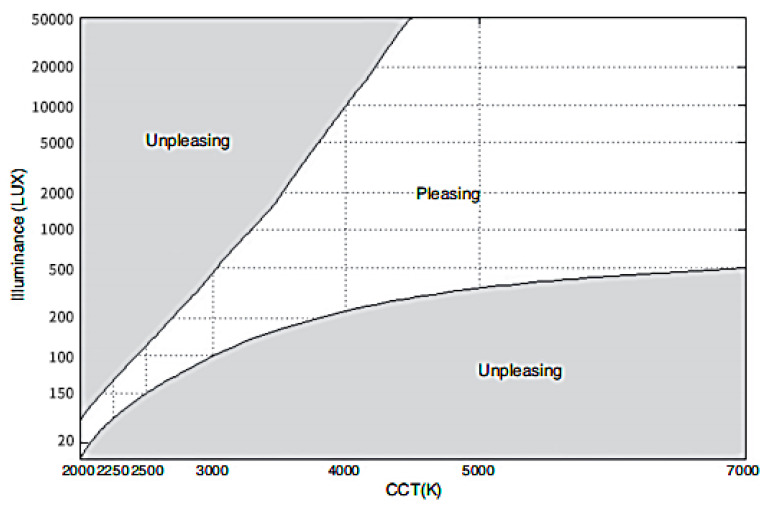
Curve of Kruithof [5].

Kruithof proposed a graph based on the relationship between CCT and illuminance in indoor lighting design to describe a comfort zone with corresponding CCT and illuminance in 1941; that is, people would feel comfortable under the specific combination of CCT and illuminance, as shown in Figure 1. Since then, scholars have carried out many experimental studies around CCT, among which Chen et al. verified Klushoff’s curve again [11,12,13]. More and more researchers have found that correlated color temperature (CCT) that is too high or too low is not popular with the observer because it makes them feel too cold or too hot [14,15]. The optimal range of CCT has different results under different exhibits, lighting layouts, and other lighting parameters, but it roughly fluctuates in a gradually specific range from 3000 K to 6000 K [16,17]. In museum exhibitions, people may prefer neutral CCT and be affected by other lighting parameters, their geographical or cultural background, and other factors.

As for research methods, researchers have tried to use virtual reality technology, often used for environmental research, to simulate real museum exhibitions [18,19,20]. For the data collection of participants, commonly used subjective assessment methods such as the Likert scale, the dual comparison method, and semantic difference are mostly used, as is the eye-tracking technology commonly used in visual perception and preference research [21,22]. Psychophysiological variables such as electrodermal activity (EDA), heart rate variability (HRV), and electroencephalogram (EEG) used in emotional measurement in other fields are rarely used in the research of museum exhibitions, but the effectiveness of these technologies in the research of perception and preference has been largely verified [23,24,25].

This paper aims to study the impact of CCT on visitors’ perception and preference in museum exhibitions, and we can more easily implement the experiment in the virtual reality environment. We will conduct a preliminary exploration of an exhibition, a unit in a museum, excluding the complex spatial factors in the real museum that are three-dimensional and complex with multiple exhibitions, so as to lay the foundation for future research. The difference between museum exhibitions and other art exhibitions is that there will be more cultural relics, especially in areas with rich history and culture. Therefore, we set up an exhibition with the theme of classical furniture as the experimental scene. We invited 50 participants to visit scenes with different CCTs and combined the psychophysiology variables and subjective evaluation of participants to explore the relationship between different CCTs of museum exhibitions and the visitors’ perception and preference. This study was designed to provide guidance for the lighting setting of museum exhibitions and focuses on participants’ psychophysiological variables that, to date, have not been studied in depth in virtual reality museum exhibitions.

## 2. Materials and Methods

An experimental study was undertaken to explore participants’ perception and preference in museum exhibitions; subjective assessment and psychophysiological variables of participants were measured in the virtual reality museum exhibitions. Various analyses were performed on the collected data. Firstly, the participants’ subjective perception and preference of correlated color temperature (CCT) were analyzed according to the results of their subjective assessment. Then, psychophysiological variables recorded the impact of the CCT on participants’ eye movement data, electrodermal activity (EDA), and heart rate variability (HRV). Subsequently, an examination was made of the sex differences and major differences in CCT perception and preference, as well as possible design guidelines for the setting of CCT lighting in museum exhibitions.

### 2.1. Participants

Fifty participants—30 females and 20 males—were recruited for this experiment. The participants were all university students, aged 20–30 years. Among them, 31 participants were science and engineering majors, and 19 were humanities and arts majors. There are four requirements for participants: 1. The corrected vision is normal. If the participants have myopia or astigmatism, they will need to wear glasses during the experiment. 2. Normal color vision; no color weakness or color blindness. 3. Do not take drinks or drugs that may affect mental state, such as alcoholic drinks, coffee, tea, cigarettes, tranquilizers, etc., within 24 h of the experiment. 4. Have enough rest before the experiment to ensure good condition [26].

We used the “Color Vision Checklist” (Second Edition) to test the participants’ color vision. According to the instructions, the first 2–8 pages of the book can be used as a test to distinguish between normal and abnormal vision, and any three pages can be identified, i.e., the color vision is normal [27]. All participants had normal corrected vision, no astigmatism, and no physical or mental disorders; one person failed the color vision test, and one person’s eye movement data was invalid. Finally, 29 male and 19 female participants were included in the data analysis. They all had previous experience visiting museum exhibitions. All the participants completed their experimental sessions successfully. Verbal and written informed consent were obtained from all the participants before they participated in the experiment.

### 2.2. Experimental Devices and Settings

The location for the experiment was an ergonomics laboratory at Nanjing Forestry University. Participants were asked to wear a helmet-mounted eye tracker and physiological sensors to complete tasks. The eye tracker (Tobii Pro VR Integration, Tobii Tech., Stockholm, Sweden) allows researchers to run eye tracking studies in fully-controlled virtual environments, easily repeat research scenarios, and switch stimuli, all while keeping track of the participants’ gaze. The system has a sampling rate of 120 Hz and adopts advanced slip compensation technology, which can ensure the accuracy of data and the effectiveness of calibration. When the headwear module is offset, it allows the maximum degree of freedom without affecting the quality of eye movement data, and is suitable for most test groups, including glasses wearers. It supports 3D models as stimulation material and eye movement data acquisition and playback in Unity 3D. The physiological sensors (ErgoLAB Physio Ergonomics Recorder, a group of wearable multi-parameter comprehensive detectors, Kingfar International Inc., Beijing, China) are the wearable ergonomics recorder; one measures electrodermal activity (EDA), the other photoplethysmography (PPG), and heart rate variability (HRV) data can be obtained from it.

**Figure 2:**
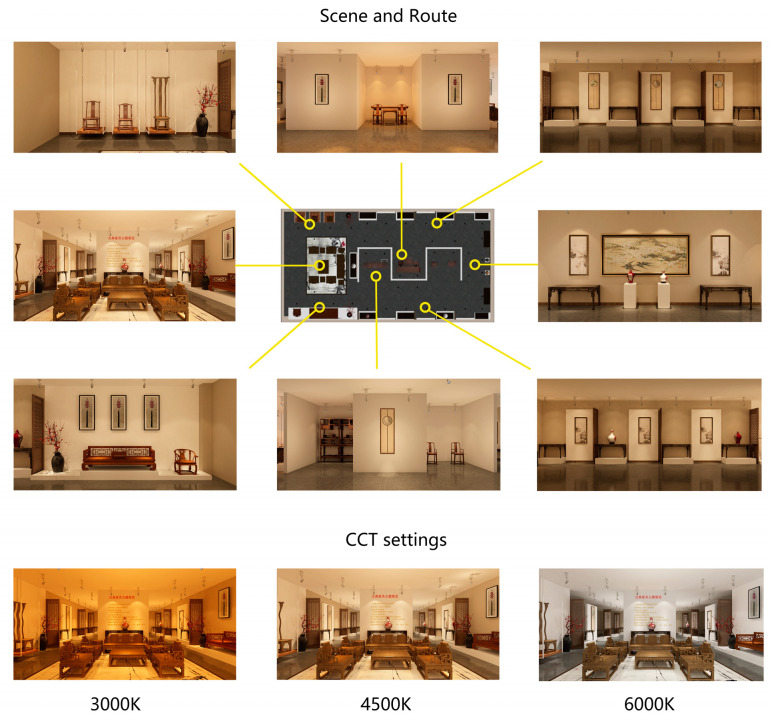
Virtual reality exhibition.

Three identical museum exhibitions with the theme of classical furniture were built by Autodesk 3D’s Max 2017. The lights were rendered in Unity 3D, where the CCT was set to 3000 K, 4500 K, and 6000 K, respectively, and the illumination was set to 6000 lm to ensure the visual brightness. In order to eliminate the interference of routes and ensure that participants can visit all exhibits, the exhibition adopts a single-line design. Participants can only visit the exhibition in clockwise or counterclockwise order. The exhibition environment of a virtual reality museum is shown in Figure 2. Model optimization and experimental debugging were conducted in Unity 3D. The experimental platform was run on Steam VR in a formal experiment. The procedure and subjective evaluation questionnaire were constructed in the Ergolab (the human–machine environment synchronization test cloud platform, Kingfar International Inc., Beijing, China).

### 2.3. Procedure

Each experimental session lasted about 40 min. The experimenter entered the basic information of the participants and helped them wear physiological sensors. Participants were asked to rest for 5 min to obtain psychophysiological variables in the resting state. Then help participants wear eye trackers. After eye-tracking calibration, participants were prompted to “be familiar with the operation in situ, the handle controls the position movement, and the body sense controls the direction. There is only one route. You can walk forward or backward freely to visit, and the visit ends when you return to the origin again”.

The participants visited the virtual reality museum exhibition freely, and the data were recorded by the Ergolab through an eye tracker and sensors. The subjective evaluation scale was filled out after visiting. After visiting all exhibitions, participants were asked to complete the preference ranking and conduct a brief interview. During the whole experiment, one experimenter was responsible for the operation of devices and the platform, and another experimenter was responsible for guiding the participants.

### 2.4. Psychophysiological Recordings

Psychophysiological variables were collected through the eye tracker and physiological sensors, which would allow us to measure indirectly the variations in states of emotion when visiting environments with different correlated color temperatures (CCTs).

The main indexes recorded from the eye tracker were average pupil diameter, saccade frequency, and blink frequency, which were used to measure visual comfort and preferences. The electrodermal activity (EDA) sensor records electrodermal activity, which is dependent on variations of sweat secreted by eccrine sweat glands in the hypodermis, and measures responses of the autonomic nervous system to timely stimuli presented to the user. The PPG sensor records the blood volume pulse waveform [28]. Heart rate variability (HRV) can be calculated from PPG data, which is associated with both sympathetic and parasympathetic nervous system activity. The original data were processed and exported by the synchronous data processing function of Ergolab.

### 2.5. Subjective Assessments

Subjective assessments are divided into two parts: a perception scale and a preference ranking. The perception scale is expanded to seven options based on the Likert scale, which allows participants to have a more detailed scoring space. The perception scale contains four dimensions: comfort and warmth are selected from visual perception, and pleasure and arousal from emotional perception. Comfort and warmth are commonly used in visual preference research. In the experiment of H-S Chen et al., the principle component analysis (PCA, a statistical technique that can be used to identify the number and nature of the independent components within a data set) was used to reduce the dimensions of visitors’ visual perception of light, and it was found that all dimensions can be summarized in two dimensions: comfort is related to colorful/dull, bright/dark, active/passive, and other dimensions; warmth is correlated with relaxation/tension and softness/hardness [13]. Psychologists A. Mehrabian and J.A. Russell proposed that people’s emotional response to environmental stimuli has three dimensions: pleasure, arousal, and control. Many scholars believed that pleasure and arousal were sufficient to represent the emotional response brought by the environment and had verified its effectiveness [29]. Therefore, in this paper, comfort and warmth were used as visual dimensions, and pleasure and arousal were used as emotional dimensions in the perception scale. After all the visiting, participants were asked to rank the three scenes according to their preferences and received brief interviews.

### 2.6. Data Processing and Analysis

All data were imported into SPSS 25 for statistical analysis, where the original data of electrodermal activity (EDA) and PPG sensors are filtered on the Ergolab. Heart rate variability (HRV) was calculated from PPG data. EDA Time Domain SC data refer to the trend of individual SC signals changing with time under the stimulation of the external environment. SC will change according to individual differences and changes in the experimental environment; therefore, all values need to be analyzed in combination with the baseline to be meaningful. According to the formula for rate of change (ROC): ROC = (measured- baseline)/baseline, the mean value of the SC signal was analyzed. Normal distribution and homogeneity of variance were evaluated by a Shapiro–Wilk test and Levene’s test, and a corresponding one-way ANOVA test and Mood’s median test were conducted accordingly. The differences between different groups of sexes and majors were assessed using a chi-squared test [30].

We carried out statistical analysis on the scores of the subjective evaluation scale in three virtual reality museum exhibitions. The subjective evaluation of each dimension was investigated by a Friedman test, respectively. We analyzed the frequency of CCT preference ranking data and assigned different weights. The most conventional method of assignment is to score the data in reverse: three points for the first place, two points for the second place, and one point for the third place. After processing the code, we obtained the results of the descriptive statistics.

## 3. Results

The experimental data are divided into two parts: psychophysiological variables and subjective assessment. The statistical analysis of the data produced the following results.

### 3.1. Psychophysiological Variables

The average levels of eye movement, electrodermal activity (EDA), and heart rate variability (HRV) of participants were obtained.

#### 3.1.1. Significance Test

Table 1 provides an overview of the significance tests of all psychophysiological variables from devices and sensors. A one-way ANOVA was used for the data that conformed to the normal distribution and had the same variance, and Mood’s median test was used for the other data and the Friedman test.

The non-significance of some data was different from the expected assumptions that SC data was sensitive to feelings. Therefore, the key psychophysiological variables of eye movement, EDA, and HRV were taken as separate variables to perform a Friedman test on the data of different correlated color temperature (CCT) groups. The results were similar to those of Mood’s median test, except that the significance of the mean pupil diameter increased while the significance of LF/HF decreased slightly. Comprehensive test results showed that mean pupil diameter and minimum pupil diameter were significant (*p* = 0.03), while LF/HF had borderline statistical significance (*p* = 0.05).

#### 3.1.2. Eye Movement and Heart Rate Variability

Among the measured psychophysiological indicators, the average pupil diameter and the minimum pupil diameter in the eye movement data, and LF/HF in heart rate variability (HRV) have statistical significance. Because the mean pupil diameter and minimum pupil diameter did not conform to the normal distribution, the percentile or median value would better reflect the data distribution trend than the mean value. We drew the mean pupil diameter and minimum pupil diameter of the participants into a boxplot, and the median of LF/HF into a line chart to observe the data distribution trend. 

**Figure 3:**
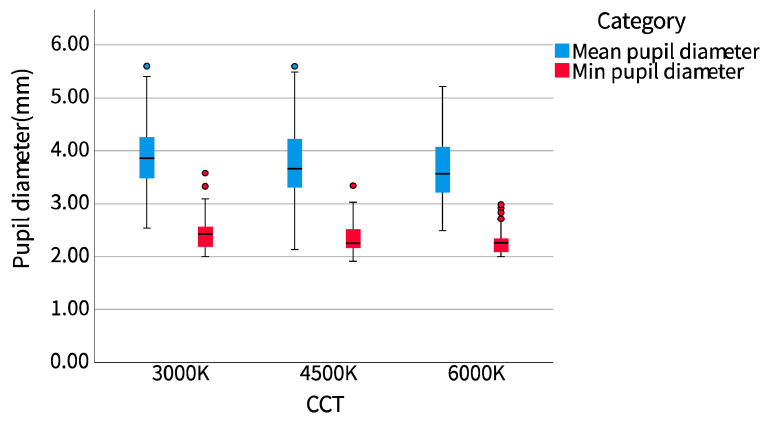
Pupil diameter.

**Figure 4:**
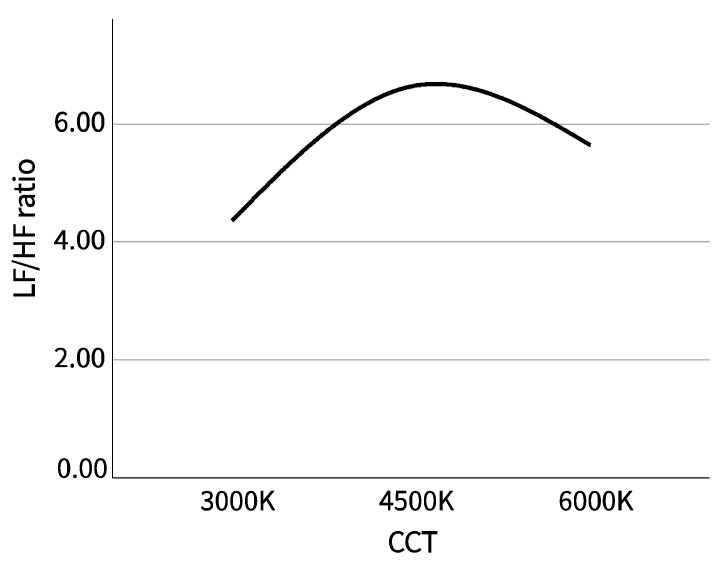
LF/HF ratio.

It could be seen from Figure 3 that the mean pupil diameter and minimum pupil diameter of the participants decreased with the increase of correlated color temperature (CCT), while LF/HF reached the highest level in the scene with CCT at 4500 K, followed by 6000 K, and the highest level was 3000 K, as shown in Figure 4. The data for the mean pupil diameter at 4500 K CCT were more dispersed, indicating that the individual differences of the participants in the scene were greater. The data for the minimum pupil diameter at 6000 K CCT were significantly concentrated, and the median of the minimum pupil diameter was significantly offset in different scenes.

#### 3.1.3. Sex and Major Differences in Psychophysiological Variables

Next, differences were examined based on the participants’ sex and major.

The significant psychophysiological variables such as pupil diameter and LF/HF were weighted by case, and then CCT-sex and CCT-major were used as cross factors to obtain chi-square test results. The subjective evaluation scale was tested by a Friedman test according to sex and major, and the preference ranking was analyzed by frequency.

**Figure 5:**
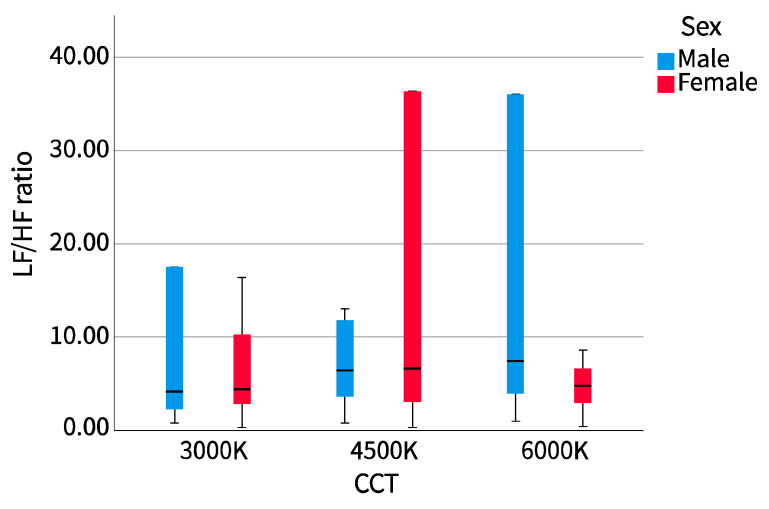
LF/HF ratio boxplot (sex).

**Figure 6:**
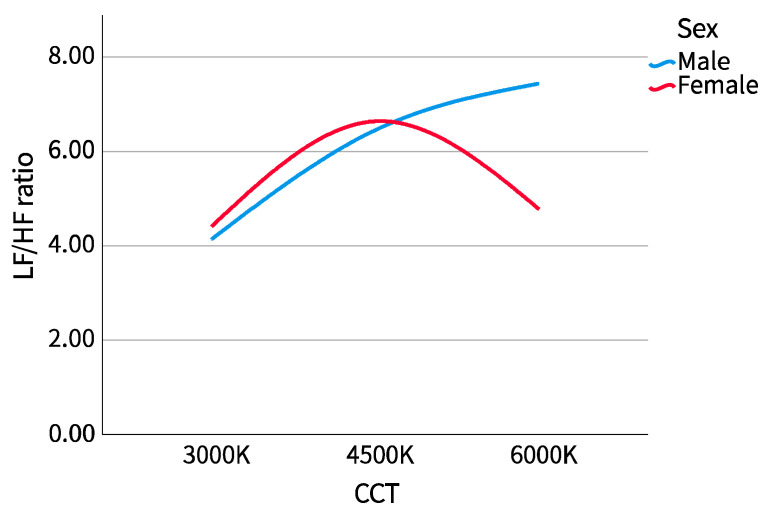
LF/HF ratio line chart (sex).

**Figure 7:**
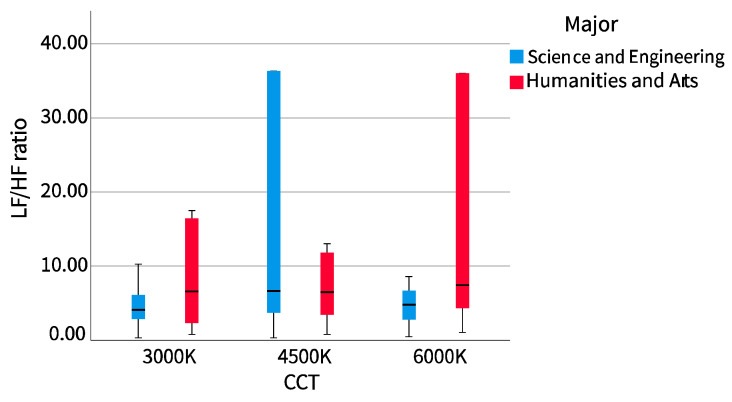
LF/HF ratio boxplot (major).

**Figure 8:**
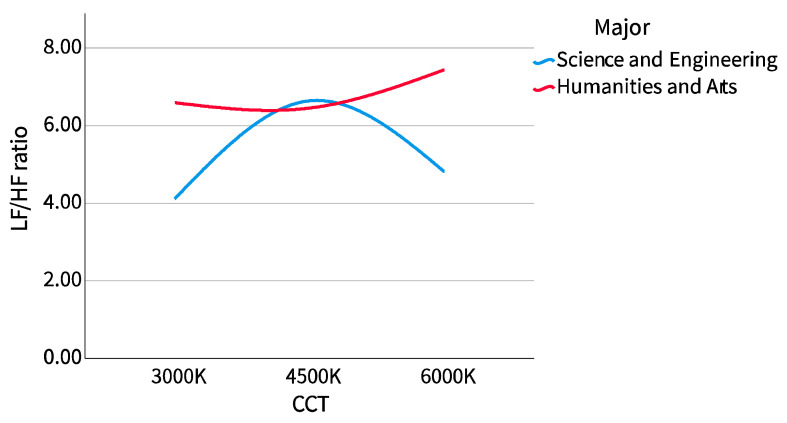
LF/HF ratio line chart (major).

The results of the cross-examination of CCT with sex and major are shown in Table 2. The mean pupil diameter showed no difference in sex or major, while LF/HF showed the opposite. We drew the LF/HF ratio of different sexes and majors into a boxplot to observe the distribution range and dispersion and drew a line chart to observe the trend, as shown in Figure 5, Figure 6, Figure 7 and Figure 8.

##### 1. Sex

From the perspective of the distribution range of data, the dispersion of LF/HF for male and female participants at different correlated color temperatures (CCT) was opposite. With the increase in CCT, the LF/HF ratio of male participants was first dispersed, then concentrated, and then dispersed. It was most concentrated at 4500 K CCT, then dispersed to both sides, and most dispersed at 6000 K CCT. Alternatively, the LF/HF ratio of female participants was first concentrated, then dispersed, and then concentrated with the increase of CCT, and the dispersion was maximum at 4500 K CCT, then focused on both sides, and focused most at 6000 K CCT. From the median trend, male and female participants at 3000 K and 4500 K CCTs were similar, showing an upward trend, but female participants’ LF/HF ratio was slightly higher than that of male participants. Female participants’ LF/HF ratio decreased significantly at 6000 K CCT, while the trend among male participants continued to rise.

##### 2. Major

The dispersion of the LF/HF ratio of science and engineering participants showed a similar trend to that of female participants. With the increase of correlated color temperature (CCT), the LF/HF ratio first concentrated, then dispersed, and then concentrated. At 4500 K CCT, the dispersion was the largest and then concentrated on both sides. The LF/HF ratio of the humanities and arts participants showed a similar trend to that of the male participants. With the increase of the CCT, it was first dispersed, then concentrated, and then dispersed. It was most concentrated at 4500 K, then dispersed to both sides, and most dispersed at 6000 K. The science and engineering participants also showed a similar trend with female participants. The LF/HF ratio increased from 3000 K to 4500 K CCTs and then decreased from 4500 K to 6000 K CCTs. The LF/HF ratio of the humanities and arts participants changed very gently, first decreasing with the increase of CCT, then decreasing to its lowest point at 4500 K, and then gradually increasing.

### 3.2. Subjective Assessments

The results of the perception scale and preference ranking were analyzed.

#### 3.2.1. Perception Scale

Taking into account that the variables do not meet the normality criterion, the perception scale was tested by a Friedman test, as shown in Table 3. There were significant differences in warmth, comfort, and pleasure, while arousal was not statistically significant.

**Figure 9:**
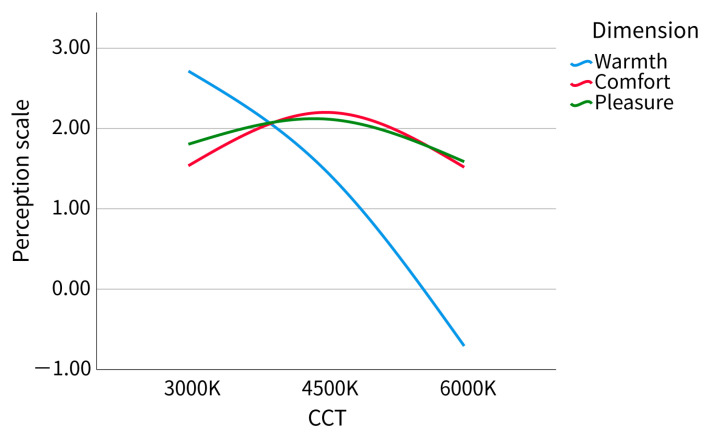
Perception scale.

We constructed a line chart as shown in Figure 9. The warmth score decreased with the increase in correlated color temperature (CCT), and the trend of comfort and pleasure scores was the same. The scene with CCT at 4500 K was the most comfortable and pleasant for participants, followed by the scene with CCT at 3500 K, and finally the 6000 K scene, but the overall score is positive.

The cross-examination results of subjective evaluation are shown in Table 4. There was no significant sex difference in all dimensions. The difference caused by major was only reflected in the comfort score.

**Figure 10:**
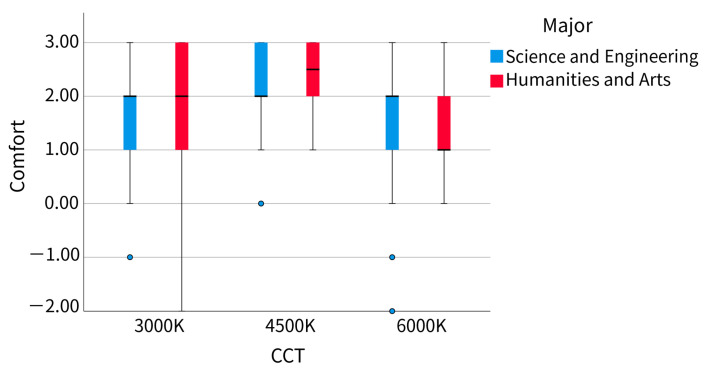
Comfort score boxplot (major).

**Figure 11:**
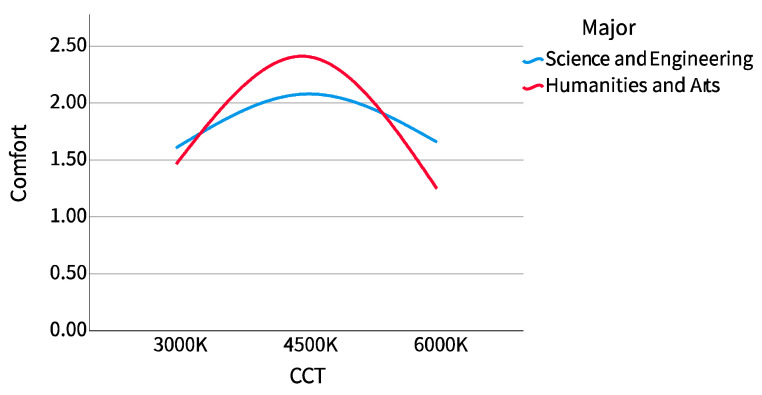
Comfort score line chart (major).

It can be seen from Figure 10 that the dispersion of comfort scores did not change much, no matter under what correlated color temperature (CCT) or for participants of different majors. In particular, the median of science and engineering participants was almost the same under different CCTs, except that the comfort scores of humanities and arts participants at the 3000 K CCT are more dispersed than those of other data groups. It can be seen from Figure 11 that participants of all majors believe that the comfort of 4500 K CCT was the highest, but the trend among science and engineering participants was clearly more gentle. The comfort score of humanities and arts participants was in the range of 4500 K, 3000 K, and 6000 K from high to low, while that of science and engineering participants was 4500 K, 6000 K, and 3000 K.

#### 3.2.2. Preference Ranking

Descriptive statistics of frequency analysis of preference ranking are shown in Table 5. In general, the scene with the highest correlated color temperature (CCT) at 4500 K ranks first, which was consistent with the results of the perception scale and psychophysiology data. The score of the 6000 K scene was slightly higher than that of the 3000 K scene, but in fact, the two scores were very close. The standard deviation of the ranking score of the 4500 K scene was the smallest, while the standard deviation of the ranking score of the 6000 K scene was the largest. That is to say, participants’ likes and dislikes of the 4500 K scene were more consistent and concentrated, while for the 6000 K scene, participants’ ranking was more decentralized. The comfort and pleasure of the 3000 K scene were higher than those of the 6000 K scene, but the participants were still relatively more inclined toward the 6000 K scene, which indicated that the preference choice is not always consistent with the pleasure and comfort. Finally, combined with the interview after the experiment, it was found that participants’ actual feelings would be stronger than their subjective scores. For example, the participants feel particularly warm but generally choose two instead of three, which was particularly obvious in the evaluation dimension that can reflect subjective good and bad. Participants will not choose negative scores in most cases, even if they feel uncomfortable. Therefore, participants’ real subjective evaluation may be more discrete than the claimed scores.

All the grouped participants by sex and major preferred 4500 K CCT, and the preference ranking results of male and female participants were consistent with those of the overall participants, in the order of 4500 K, 6000 K, and 3000 K. The preference ranking results of the science and engineering participants were also consistent with the overall results, but the humanities and arts participants preferred 3000 K CCT to 6000 K CCT.

**Figure 12:**
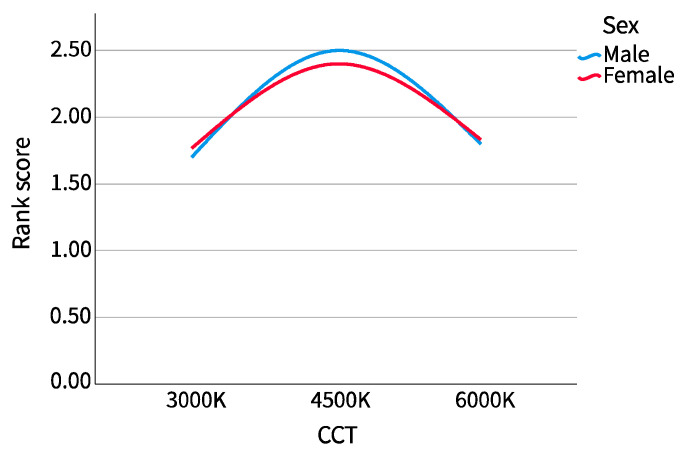
Preference ranking (sex).

**Figure 13:**
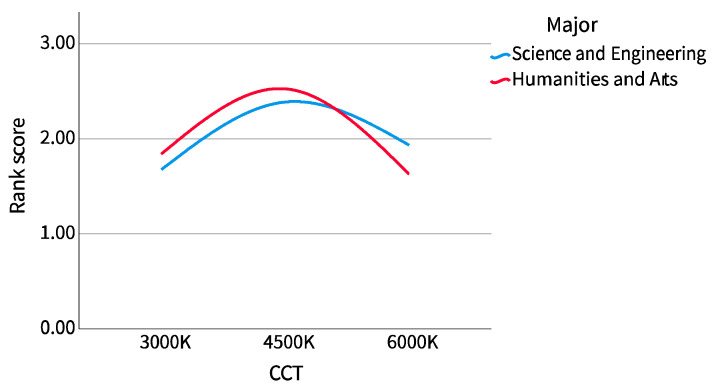
Preference ranking (major).

As shown in Figure 12 and Figure 13, the mean score after reverse scoring is plotted as a line chart according to sex and major. It can be seen that in sex grouping, there was little difference between male and female participants, but the scoring trend of female participants was slightly flat. In the major grouping, the overall difference was not great, but the scores of the humanities and arts participants dropped more in the range of 4500 K to 6000 K CCT.

## 4. Discussion

This paper aims to study the impact of correlated color temperature (CCT) on visitors’ perception and preference in virtual reality museum exhibitions that might improve the settings of museum lighting. We examined subjective assessment and psychophysiological variables of participants in museum exhibitions with different CCTs, such as perception, preference, eye movement, electrodermal activity (EDA), and heart rate variability (HRV). The guidelines will be based on both the subjective and psychophysiological responses of participants. The study findings make significant contributions at the methodological and results levels.

### 4.1. Correlated Color Temperature and Subjective Assessment

In the results of subjective assessment, the warmth decreased with the increase in correlated color temperature (CCT), while the comfort, pleasure, and preference ranking scores increased first and then decreased. The trend of warmth verified previous studies that the feeling of cold and heat changes with the change of CCT [6]. The increase in CCT does not lead to a linear increase in comfort, which is consistent with previous research results [31]. The scores for pleasure and comfort were positive, which partially conforms to Kruithof’s curve. The fixed lighting intensity in this experimental scene is 6000 lm ≈ 6000 lx. According to Kruithof’s curve, 3000 K clearly does not belong to the comfort zone, which is different from our experimental results. Although 4500 K and 6000 K belong to the comfort zone, our results showed that there is a significant difference in the degree of comfort between the two scenes. The uncomfortable zone on the Kruithof curve was rated as comfortable by participants in our experiment (average score 1.5 in the range of −3 to 3), which may be because our participants do not tend to score negatively unless they feel extremely uncomfortable. Comfort evaluation is not neutral, not the same as warmth evaluation without praise or derogation, though the experimenter emphasized that participants should only score according to their real feelings. Of course, it is also possible that factors other than CCT and illumination lead to different results. For the difference in comfort, this is not covered by the Kruithof curve; that is, different color temperatures are evaluated as comfortable, but the degree of comfort will also be different.

Participants were most inclined to the scene with CCT at 4500 K, followed by 6000 K, and finally 3000 K. It is similar to some research in recent years, which found that the arrangement of supercooled or overheated lights was not popular with visitors, so visitors actually preferred the CCT range of about 4500 K. There were also many similar studies in other building spaces [31,32]. The trend of comfort and pleasure was similar, but it showed that a 3000 K scene is more comfortable and pleasant than a 6000 K scene, which was different from the results of preference ranking. It indicated that participants’ preferences are not only related to comfort and pleasure. For example, participants’ perceptions of temperature in the environment may also have an impact on CCT preferences. Some studies have shown that people such as warm feelings in cold climates [33,34,35]. Our experiment was conducted in eastern China in October (room temperature of about 15 °C); therefore, it may lead to participants’ preference for neutral CCT.

### 4.2. Correlated Color Temperature and Psychophysiological Variables

The study measured three psychophysiological variables: eye movement, electrodermal activity (EDA), and heart rate variability (HRV) to explore participants’ correlated color temperature (CCT) perception and references, and the final results showed that EDA were not significantly different in scenes with different CCTs. As a common index of emotional arousal, EDA is rarely used in the research about CCT in the museum. Unfortunately, there was no significant correlation between EDA and CCT in our experiment. The possible reason is that the difference between our experimental stimuli is not enough to cause significant changes in EDA, while the HRV, which is also commonly used in emotional measurement, has an evident discrepancy in our study, which may indicate that the threshold of stimuli that can cause obvious changes in EDA is higher than that of the HRV. The adoption of eye-tracking was due to its role in the study of visual preference, which can reflect visual cognitive and psychological processes [36,37]. Kaihong Zhang et al. showed that the change in pupil diameter was negatively correlated with visual comfort in the study of stereo image chromaticity [21]. In our experiment, the pupil diameter decreased with the increase of CCT, which is only consistent with the warmth in the perception scale, while comfort, pleasure, and preference ranking scores all increased first and then decreased with the increase of CCT. There are three possible reasons. One is the deviation between the subjective assessment and the changes in the objective psychophysiological variables among participants. The other is the difference in experimental stimulation. The influence of CCT changes and chromaticity changes on the participants is different. The third is the limitations of Zhang et al.’s experiment. The negative correlation between pupil diameter and comfort may exist within a range. After all, the pupil diameter cannot be infinitely reduced or increased.

Among the indicators of HRV, LF/HF represents the energy ratio of low frequency and high frequency, reflecting the balance of sympathetic and parasympathetic nerves, and is related to the arousal level [38]. In our research results, the trend of the LF/HF ratio showed that the arousal level of participants was highest at 4500 K CCT, followed by 6000 K, and finally 3000 K, which was consistent with the trend of preference ranking, but there was no significant difference in the subjective assessment of arousal by participants, which is contrary to the research results of Marin Morales et al., that is, the direct virtualization of the real environment may cause psychological arousal by self-report, but it may not trigger the same cardiovascular changes as the arousal [39]. The difference in the significance of the LF/HF ratio may indicate that evident heart rate variability (HRV) changes will occur only when the stimulation intensity for the human body reaches a certain threshold in the virtual reality environment. It is surprising that there is no statistical difference in the arousal of participants’ self-assessment. From a numerical point of view, the arousal degree scores of the three scenarios are indeed very close. It may indicate that participants were unaware of the changes in the LF/HF ratio; that is to say, our experimental stimulation was not enough to make participants aware of the changes in arousal. 

### 4.3. Sex and Major Differences

There are significant sex differences in participants’ LF/HF ratio, which is consistent with past cognition that men and women often have different visual preferences [31,40]. Huang, Z et al. found that people’s preferences for color have significant sex differences under some lighting conditions, especially for the cases with higher CCTs (5000 K and 6500 K). Our experimental results also found that the LF/HF ratio has more clear sex differences under high correlated color temperature (CCT) conditions. However, there is no such difference in our subjective assessment, which may be because the sex difference is for CCT and color, and the dimension used in our subjective assessment is also different from the experiment of Huang et al. [41]. In comparison, female participants prefer a low CCT. The arousal level of male participants continued to rise with the increase in CCT, while female participants reached a peak at 4500 K CCT and then fell. That is to say, women prefer a warm, light environment over men. In previous studies, it was found that, compared with men, women do not like light sources with high white perception, which is related to our experimental results [42].

The LF/HF ratios of participants from different majors were significantly different, which was hardly mentioned in previous studies. In our research results, the LF/HF ratios of humanities and arts participants and science and engineering participants had opposite trends; that is, the arousal level of humanities and arts participants was the lowest at 4500 K CCT, and the arousal level rose at low CCT and high CCT, but the change was relatively gentle. Science and engineering participants had the highest arousal level at 4500 K CCT, while the arousal level at low CCT and high CCT decreased, and science and engineering participants were more sensitive to changes in arousal level. However, in the comfort and preference ranking, the data trend of science and engineering participants was more gentle than that of humanities and arts participants. Therefore, in general, the change trend of the heart rate variability (HRV) index of science and engineering participants was opposite to that of humanities and arts participants, but the trend of subjective assessment was consistent with a small gap.

### 4.4. Advantages and Limitations

For the experimental task, participants were allowed to visit the virtual reality museum exhibition and simulate the real museum exhibition experience, which not only makes the experiment simpler and easier to operate and realizes the research of the space environment in the limited experimental environment but also leaves enough time for the measurement of the participants’ psychophysiological variables in the same state. Among the psychophysiological variables measured in the experiment, HRV was highly sensitive, reflecting more information and highlighting the advantages of HRV in perception and preference research.

Despite these findings, our study still has some limitations. First, the participants of this experiment were college students, and it is not clear whether the research results are applicable to other age groups or other occupational groups. Second, the virtual reality museum exhibition was built in our experiment, but it is not clear whether the experimental results are applicable to the real environment or which parts are applicable to the real environment. Third, in the experiment, the ambient temperature may affect the participants’ perception and preference of CCT. 

## 5. Conclusions

The results of this study contribute to the correlated color temperature (CCT) settings of light in museum exhibitions in the following aspects: We used virtual reality technology to study participants’ perception and preference of CCT in museum exhibitions by combining psychophysiological variables and subjective assessment. We verified participants’ preferences for neutral CCT under high illumination conditions and female participants’ preference for lower CCT. Moreover, we found the trend in the change of the LF/HF ratio was opposite for participants of different majors (science and engineering, humanities, and arts), but the trend in subjective assessment was similar. Science and engineering participants are more inclined to high CCT (6000 K) than humanities and arts participants. Our experimental results can provide some theoretical references for practitioners when setting the lighting parameters of museum exhibitions so as to improve the satisfaction of visitors. In most cases, on the basis of not damaging cultural relics, neutral CCT is an error-free choice. In addition, practitioners can make some more detailed adjustments to CCT according to the audience of the museum exhibition. For example, women account for the majority of visitors in some exhibitions, so the CCT can be set relatively low. Or in the science and technology exhibition, the CCT can be set higher according to the theme. Further exploration is needed for the underlying reasons or principles behind these findings. In future studies, in terms of experimental participants, we may refine the population classification and screening criteria, such as age, cultural background, familiarity with the operation of virtual reality technology, and understanding of the light environment or museum exhibition. In the aspect of an experimental environment, we may compare the difference between a virtual reality museum exhibition and a real museum exhibition experience and further explore the scientific feasibility of an environment experiment using virtual reality technology. We may consider the experimental variables, controlling possible interference factors such as room temperature, and adding illumination, color rendering index (CRI), lighting mode, or other parameters in future experiments so as to provide a more comprehensive contribution to the light environment setting of the museum exhibitions.

## Figures and Tables

**Table 1:** Psychophysiology variables from all devices and sensors.

		Min	Max	Mean	Variance	Median	S-W	*p*	F-M
Eye movement	Mean Pupil diameter (mm)	2.13	5.60	3.77	0.54	3.67	0.04 ^c^**	0.03 ^a^**	0.00 ***
	Min Pupil diameter (mm)	1.00	3.58	2.37	0.12	2.30	0.00 ^c^**	0.03 ^a^	-
	Max Pupil diameter (mm)	2.54	6.96	5.00	0.72	4.95	0.00 ^c^**	0.28 ^a^	-
	Blink Count (N)	1.00	310.00	25.52	1032.11	16.50	0.00 ^c^**	0.57 ^a^	-
	Blink Frequency (N/s)	0.01	1.20	0.16	0.03	0.11	0.00 ^c^**	0.49 ^a^	0.22
	Saccade Count (N)	324.00	2312.00	974.35	156,614.59	900.65	0.00 ^c^**	0.92 ^a^	-
	Saccade Frequency (N/s)	3.49	8.76	6.35	0.86	6.33	0.08	0.24 ^b^	-
	Total Saccade Time (s)	15.81	120.09	47.90	385.61	44.88	0.00 ^c^**	0.92 ^a^	-
EDA	Time Domain Phasic (μS)	−0.91	25.72	1.13	11.28	0.22	0.00 ^c^**	0.73 ^a^	-
	Time Domain SC (μS)	−0.78	2.25	0.28	0.33	0.15	0.00 ^c^**	0.92 ^a^	0.35
	Time Domain Tonic Data (μS)	−0.56	2.22	0.29	0.34	0.15	0.00 ^c^**	0.92 ^a^	-
PPG	Mean IBI (ms)	498.13	977.64	708.69	9332.86	692.84	0.01 ^c^**	0.92 ^a^	0.92
	Mean HR (bpm)	61.00	120.00	86.18	133.21	86.50	0.29	0.85 ^b^	-
	SDNN (ms)	12.02	774.60	71.63	6433.08	48.56	0.00 ^c^**	0.38 ^a^	-
	RMSSD (ms)	6.37	1026.73	72.58	10,399.88	43.56	0.00 ^c^**	0.79 ^a^	-
	SDSD (ms)	6.38	1030.79	72.82	10,485.51	43.68	0.00 ^c^**	0.79 ^a^	-
	pNN50 (%)	0.38	66.15	16.72	291.38	9.49	0.00 ^c^**	0.57 ^a^	-
	Total Power (ms^2^)	110.70	34,523,710.93	265,369.20	7,959,389,245,633.53	2069.46	0.00 ^c^**	0.19 ^a^	-
	LF/HF ratio	0.31	36.34	3.88	24.86	2.71	0.00 ^c^**	0.05 ^a^*	0.10

^a^ Mood’ s median test, ^b^ one-way ANOVA test, ^c^ Shapiro Wilk test. *** *p* < 0.01, ** *p* < 0.05, * *p* = 0.05.

**Table 2:** Cross examination of psychophysiological variables.

		Mean Pupil Diameter			LF/HF Ratio		
		Value	Freedom	*p*	Value	Freedom	*p*
Sex	Pearson chi square	0.17	2	0.92	11.61	2	0.00 ***
	Likelihood ratio	0.18	2	0.92	11.65	2	0.00
	Linear correlation	0.11	1	0.75	11.15	1	0.00
	Number of valid cases	565			582		
Major	Pearson chi square	0.01	2	0.99	9.58	2	0.01 **
	Likelihood ratio	0.01	2	0.99	9.61	2	0.01
	Linear correlation	0.00	1	0.98	3.82	1	0.05
	Number of valid cases	566			584		

** *p* < 0.05, *** *p* < 0.01.

**Table 3:** Friedman test of perception scale.

	Number	Chi-Square	Freedom	*p*
Warmth	50	84.17	2	0.00 ***
Comfort	50	18.24	2	0.00 ***
Pleasure	50	10.49	2	0.01 **
Arousal	50	2.20	2	0.33

*** *p* < 0.01, ** *p* < 0.05.

**Table 4:** Cross examination of perception scale by sex and major.

		Warmth			Comfort			Pleasure		
		Value	Freedom	*p*	Value	Freedom	*p*	Value	Freedom	*p*
Sex	Pearson chi square	0.28	2	0.87	0.28	2	0.87	0.28	2	0.87
	likelihood ratio	0.28	2	0.87	0.28	2	0.87	0.28	2	0.87
	Linear correlation	0.00	1	0.98	0.00	1	0.98	0.00	1	0.98
	Number of valid cases	217			217			217		
Major	Pearson chi square	0.34	2	0.84	6.92	2	0.03 **	2.83	2	0.24
	likelihood ratio	0.34	2	0.85	7.12	2	0.03	2.84	2	0.24
	Linear correlation	0.33	1	0.57	6.75	1	0.01	2.81	1	0.09
	Number of valid cases	217			215			203		

** *p* < 0.05.

**Table 5:** Frequency analysis results.

	Option	One Choice Quantity	Second Choice Quantity	Three Choice Quantity	Min	Max	Mean	SD	Ranking
Overall	4500 K	27	18	5	1	3	2.44	0.67	1
	6000 K	13	15	22	1	3	1.82	0.83	2
	3000 K	10	17	23	1	3	1.74	0.78	3
Male	3000 K	4	6	10	1	3	1.70	0.80	3
	4500 K	12	6	2	1	3	2.50	0.69	1
	6000 K	4	8	8	1	3	1.80	0.77	2
Female	3000 K	6	11	13	1	3	1.77	0.77	3
	4500 K	15	12	3	1	3	2.40	0.67	1
	6000 K	15	12	3	1	3	1.83	0.87	2
Science and Engineering	3000 K	5	11	15	1	3	1.68	0.75	3
	4500 K	16	11	4	1	3	2.39	0.72	1
	6000 K	10	9	12	1	3	1.94	0.85	2
Humanities and Arts	3000 K	5	6	8	1	3	1.84	0.83	2
	4500 K	11	7	1	1	3	2.53	0.61	1
	6000 K	3	6	10	1	3	1.63	0.76	3

## Data Availability

Not applicable.
